# Efficacy of Conservative Approaches on the Physio-Mechanical Properties of Caries Lesions Remineralized with Silver Diamine Fluoride 

**DOI:** 10.3290/j.ohpd.c_2505

**Published:** 2026-03-30

**Authors:** Sarah S. Al-Angari, Ali Alrahlah

**Affiliations:** a Sarah S. Al-Angari Associate Professor, Department of Restorative Dental Science, Collage of Dentistry, King Saud University, Riyadh, Saudi Arabia. Conceptualization, methodology, formal analysis, validation, wrote original draft, reviewed and edited the manuscript.; b Ali Alrahlah Professor, Department of Restorative Dental Science, College of Dentistry, King Saud University, Riyadh, Saudi Arabia; Engineer Abdullah Bugshan Research Chair for Dental and Oral Rehabilitation, College of Dentistry, King Saud University, Riyadh, Saudi Arabia. Formal analysis, validation, wrote original draft, reviewed and edited the manuscript.

**Keywords:** dental caries, enamel microabrasion, shear bond strength, silver diamine fluoride, surface roughness, toothbleaching.

## Abstract

**Purpose:**

Silver diamine fluoride (SDF) is a proven caries-arresting agent, but its clinical acceptance is limited by the black staining of treated lesions. This purpose of this study was to evaluate the effects of potassium iodide (KI), bleaching, microabrasion, and their combinations on the average surface roughness (Ra) and shear bond strength (SBS) of SDF-stained remineralized caries-like lesions (s-RCLs).

**Materials and Methods:**

Human enamel slabs were demineralized and randomized into six groups (n = 17): G1, no treatment; G2, treated with 38% SDF to create s-RCLs; G3, s-RCLs+KI; G4, s-RCLs+KI+bleaching; G5, s-RCLs+KI+microabrasion, and G6, s-RCLs+KI+microabrasion+bleaching. Ra and SBS were measured and analyzed using ANOVA and Tukey’s test (α = 0.05).

**Results:**

Demineralization statistically significantly increased Ra (p ≤ 0.003). Post-treatment, G5 exhibited the highest Ra (p < 0.001) and SBS (p ≤ 0.010), while G4 had the lowest SBS (p ≤ 0.018). G6 demonstrated Ra comparable to control groups and an average SBS, indicating a favorable balance between surface smoothness and bonding.

**Conclusion:**

Our findings suggest that the combined use of KI, microabrasion, and bleaching achieved the least statistically significant Ra while maintaining an average SBS, providing superior functional outcomes compared with bleaching or microabrasion alone, making it a promising conservative approach for managing SDF-treated lesions.

Contemporary advancements in diagnostic modalities and dental materials have driven the development of minimally invasive approaches, aligning with the growing emphasis on conservative esthetic dentistry.^29^ Active caries lesions can be arrested through remineralizing techniques and materials such as sodium fluoride, casein phosphopeptides, and silver diamine fluoride (SDF).^2,32,34^

SDF is a minimally invasive therapeutic agent increasingly recognized for halting dental caries.^4^ Chemically, SDF comprises silver ions, which function as an antibacterial agent, fluoride ions, promoting remineralization, and ammonia, which is included for solution stabilization,^16,20^ demonstrating superior results to sodium fluoride in enhancing the mineral density. The application of 38% SDF, containing 25% silver, 8% ammonia, and 5% fluoride, has demonstrated efficacy and safety in arresting active caries lesions.^35^ Consequently, with periodic application, they become less prone to future demineralization.^8^ However, a relevant clinical challenge associated with SDF is the unesthetic blackish discoloration of remineralized caries lesions due to silver-phosphate surface precipitation.^6,35^ Conventional management to remove these stains often necessitates the invasive removal of sound, highly mineralized tooth structures to improve esthetics.^6^

Other conservative approaches, such as the application of saturated potassium iodide (KI), bleaching agents, and microabrasion techniques, have been suggested to mitigate SDF-induced discoloration to minimize the use of restorations.^7,8,25^ However, the resultant chemical precipitates from the KI, the enamel morphological surface changes induced by bleaching agents, and microabrasion techniques may compromise the average surface roughness and bond strength. The extant literature primarily focuses on the color outcome of SDF applications. However, an important gap persists in understanding SDF’s impact on the structural integrity and restorative potential of remineralized tissues after using such treatments. The novelty of this research lies in shifting the focus from purely esthetic improvements to the physiomechanical performance of SDF-treated enamel. To our knowledge, this is the first study that systematically examines how KI, bleaching, and microabrasion, alone or in combination, influence both the average surface roughness and bonding strength, thereby providing clinically relevant insight into the balance between esthetic management and functional integrity. The study aimed to investigate the efficacy of conservative approaches (KI, at-home bleaching, microabrasion, and their combination) on the average surface roughness and shear bond strength of artificially created caries-like lesions treated with SDF. The null hypothesis states that there would be no statistically significant difference in the conservative approaches on the average surface roughness and shear bond strength of artificially created caries-like lesions treated with SDF.

## MATERIALS AND METHODS

### Experimental Design

Artificial caries-like lesions were created on human enamel and remineralized using SDF to produce stained-remineralized caries-like lesions (s-RCLs). The study investigated the physiomechanical properties of different conservative approaches at six levels. G1: negative control (demineralized/no treatment); G2: positive control (s-RCLs); G3: s-RCLs + KI; G4: s-RCLs + KI + at-home bleaching protocol; G5: s-RCLs + KI + microabrasion; G6: s-RCLs + KI + microabrasion + at-home bleaching protocol (Table 1).^1^ Prior to the study, power analysis indicated that a sample size of 17 specimens per group provides 90% power to detect an effect size of 0.8 at a statistical significance level of 0.05. The study outcomes were surface roughness (Ra, µm) measured by a profilometer at three time points (baseline, after demineralization, and after treatment), and shear bond strength (SBS, MPa) assessed after treatment using a universal testing machine.

**Table 1:** Group definitions based on the treatment protocol used

Groups	Treatment protocol
G1	Negative control (demineralization / no treatment)
G2	Positive control (s-RCL treated with 38% SDF)
G3	s-RCL + KI
G4	s-RCL + KI + at-home bleaching
G5	s-RCL + KI + microabrasion
G6	s-RCL + KI + microabrasion + at-home bleaching
s-RCL: stained-remineralized caries-like lesion; SDF: silver diamine fluoride; KI: potassium iodide solution; at-home bleaching: 15% carbamide peroxide bleaching gel; microabrasion: 6.6% hydrochloric acid/pumice.	

### Specimen Preparation

The experimental units were enamel slabs (4 × 4 × 2 mm) from human molars, sound and free of cracks or defects. One hundred two (102) specimens were selected after approval by the Institutional Review Board (IRB# E-19-3836). Specimens were sectioned from the buccal and lingual area using a low-speed diamond saw (Isomet, Buehler; Lake Bluff, IL, USA) then embedded in acrylic resin blocks (10 × 10 × 8 mm; Varidur, Buehler). They were sequentially ground flat using 500-, 1200-, 2400-, and 4000-grit silicon carbide grinding papers (MD Fuga, Struers; Ballerup, Denmark), polished with a 1-µm diamond slurry (Struers), then sonicated in a detergent solution (Micro-90, International Products; Burlington, NJ, USA). After polishing, the specimens were placed under running deionized water for 3 min, then stored in a refrigerator at approximately 100% relative humidity at 4°C (Kenmore, Whirlpool; Benton Harbor, MI, USA) until use.

#### Production of caries-like lesions 

Enamel caries-like lesions were induced by immersing the specimens in a demineralizing solution, following the methodology of Lippert et al.^27^ All specimens were demineralized for seven days in a solution of 0.1 M lactic acid, 4.1 mM calcium chloride dihydrate (CaCl_2_2H_2_O), 8.0 mM potassium dihydrogen phosphate (KH_2_PO_4_) and 1.0% w/v carboxymethylcellulose (Sigma-Aldrich; St Louis, MO, USA) with pH adjusted to 5.0 using potassium hydroxide (KOH) at 37°C. This demineralizing solution was neither stirred nor replenished during the demineralization period. The lesion exhibited the characteristics of an incipient caries lesion, having an opaque appearance due to mineral loss caused by a change in the refractive index, with a relatively intact outer surface and an increased volume in the subsurface lesion body due to mineral loss.

#### Staining and treatment

Following demineralization, specimens were randomly assigned to one of six treatment groups (n = 17 per group), as illustrated in Table 1. Group 1 (negative control) specimens were demineralized and received no treatment. Group 2 (positive control) was treated with 38% SDF (SDF, Advantage Arrest, Elevate Oral Care LLC; West Palm Beach, FL, USA) for two minutes^12^ to create s-RCLs. Group 3: s-RCLs were treated immediately with a saturated KI solution. The KI solution (2.36 mol/l) was prepared in the laboratory using distilled water and KI powder (Sigma-Aldrich).^1^ KI creamy white solution was brushed on the surface and removed when the solution’s reaction products became clear. It was washed off with copious distilled water, after which the surface was dried. In group 4, s-RCLs were treated with KI solution followed by an at-home bleaching protocol using 15% carbamide peroxide. In group 5, s-RCLs were treated with KI solution followed by microabrasion using 6.6% hydrochloric acid/pumice. In group 6, s-RCLs were treated with KI solution, followed by microabrasion, then an at-home bleaching protocol.

#### Dental microabrasion test

The microabrasion procedure, performed on groups G5 and G6 prior to bleaching, involved the application of a 6.6% hydrochloric acid microabrasive slurry (Opalustre, Ultradent; South Jordan, UT, USA).^3,12,33^ The slurry was applied to the surface (1.0 mm thickness) and abraded using a prophylaxis rubber cup for 1 min, followed by thorough rinsing and drying. The specimens were then polished using a fluoridated prophylaxis paste (Vigodent Coltene SA Indústria e Comércio; Rio de Janeiro, RJ, Brazil). Finally, according to the manufacturer’s instructions, a 2% neutral-pH sodium fluoride gel was applied for 4 min.^3,12^

#### Dental bleaching test

Groups G4 and G6 underwent dental bleaching using an at-home bleaching protocol using 15% carbamide peroxide (CP) (pH 6.5; Opalescence PF, Ultradent). The bleaching gel was applied to the top enamel surface (0.5–1.0 mm thick) for four hours daily in a 37°C incubator over a seven-day period.^5,8^ Following bleaching, specimens were rinsed with running distilled water for 1 min to remove residual bleaching agent, blotted dry, and stored in a moist environment at 4°C until subsequent testing.

#### Surface roughness test

The surface profile was analyzed using a 3D non-contact optical profilometer (Contour GT-X, Bruker; Billerica, MA, USA). The specimen was affixed to the automated x-y stage and scanned. The scanning parameters were set as a 5× nano-lens magnification, a 0.5 × 0.5 mm view area at the specimen center, a 1× scan speed, and 0.1 mm/s stage speed. The primary parameter analyzed was the average surface roughness (Ra), defined as the arithmetic mean of the heights of peaks and depths of valleys relative to a mean line over the measuring length. Vision 64 software (v. 5.30, Bruker) was utilized to control the instrument and perform data analysis.

#### Shear bond strength test

Two weeks after the experiment, the resin composite (Filtek Z350, 3M ESPE; St Paul, MN, USA) was incrementally applied to the enamel surface using a silicone mold (Ø: 3 mm; height: 2 mm). Each resin composite layer was light cured for 20 s using an Elipar Deep cure, single-emission peak LED (3M ESPE). The irradiance of the light was 1000 mW/cm^2^, controlled by a radiometer (Caulk Dentsply; Milford, DE, USA). After removing the mold, additional light curing was performed for 20 s on all sides of the resin cement build-up, with the light-curing tip positioned as close as possible to the specimen surface. The specimens were stored in distilled water at 37°C for 24 h.^4^ Subsequently, they were mounted on a universal testing machine (Instron 5965; Norwood, MA, USA) and subjected to shear force using a notched chisel at a crosshead speed of 0.5 mm/min until failure occurred.

### Statistical Analysis

The Shapiro-Wilk test was used to assess the normality of the data, then the Ra and SBS were analyzed using one-way ANOVA, followed by Tukey’s post-hoc test for multiple comparisons (α = 0.05). Statistical analyses were conducted using SPSS Statistics v.20 software (IBM SPSS; Armonk, NY, USA).

## RESULTS

**Fig 1:**
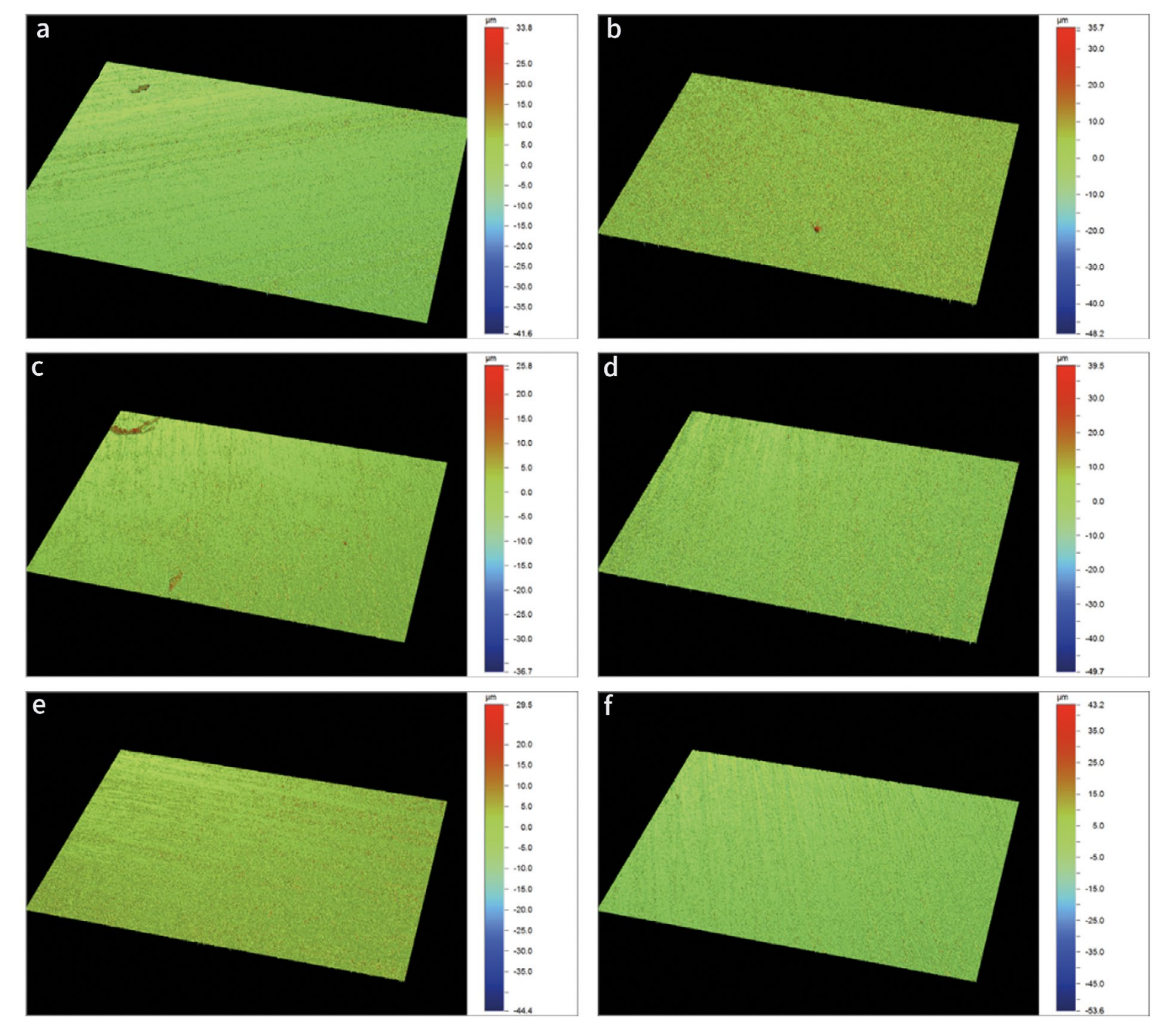

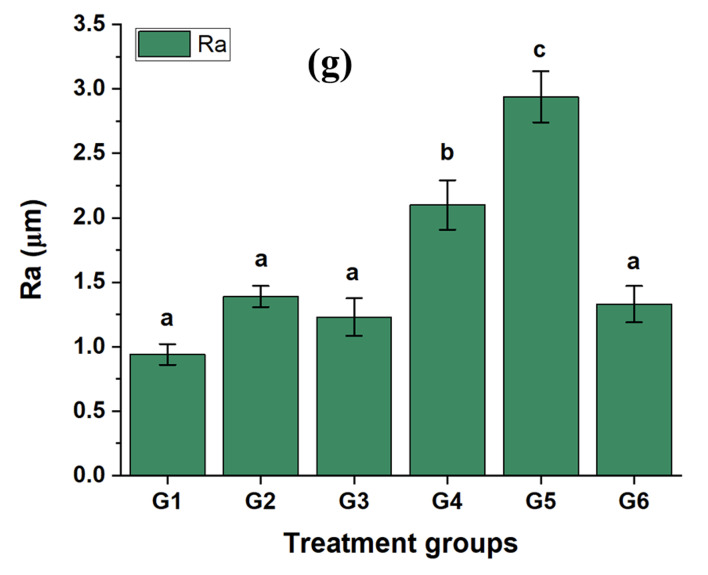
Illustration of 3D optical profilometry images of the study groups (a) G1, (b) G2, (c) G3, (d) G4, (e) G5 and (f) G6. Fig 1g: Surface roughness G1 to G6 (Ra, µm). Error bars represent the standard error of mean. Different lowercase letters indicate statistically significant differences.

The results of the Shapiro-Wilk test indicated that the data followed a normal distribution (p > 0.05), while Levene’s test confirmed the homogeneity of variances (p > 0.05). The results for surface roughness (Ra) are illustrated in Fig 1. Table 2 presents a detailed comparison of surface roughness for the treatment groups, listing the Ra values for baseline, demineralized, and treated groups. It is apparent from Fig 1 that demineralization statistically significantly increased the mean surface roughness values across all groups (p ≤ 0.003) compared to the baseline values (average: Ra-baseline = 0.42±0.1 µm, Ra-demineralization = 1.06±0.10 µm), validating the effectiveness of the demineralization process. No statistically significant differences in roughness were observed among treatment groups at baseline or after demineralization (p > 0.05). Following the treatment, G5 exhibited the highest statistically significant increase in surface roughness values (p < 0.001), followed by G4 (p ≤ 0.012). These two groups differed statistically significantly from each other (p = 0.002) and from all other groups (p ≤ 0.005). Conversely, G1, G2, G3, and G6 showed no statistically significant differences in roughness (p ≥ 0.263), though minor variations were observed.

**Table 2:** Surface roughness (Ra, µm) means (standard deviation) at baseline, after demineralization, and after the treatment protocols

Groups	Ra-Baseline	Ra-Demineralization	Ra-Treatment
G1	0.42 (0.09)^a, A^	0.95 (0.29)^a, B^	0.94 (0.33)^a, B^
G2	0.43 (0.10)^a, A^	1.19 (0.34)^a, B^	1.39 (0.34)^a, B^
G3	0.41 (0.11)^a, A^	1.03 (0.45)^a, B^	1.23 (0.60)^a, B^
G4	0.40 (0.09)^a, A^	0.97 (0.23)^a, B^	2.10 (0.79)^b, C^
G5	0.41 (0.10)^a, A^	1.08 (0.33)^a, B^	2.94 (0.82)^c, C^
G6	0.44 (0.09)^a, A^	1.15 (0.31)^a, B^	1.33 (0.58)^a, B^
Uppercase letters indicate statistically significant difference within a treatment (rows, p < 0.05) and lowercase between treatments (columns, p < 0.05).			

**Fig 2:**
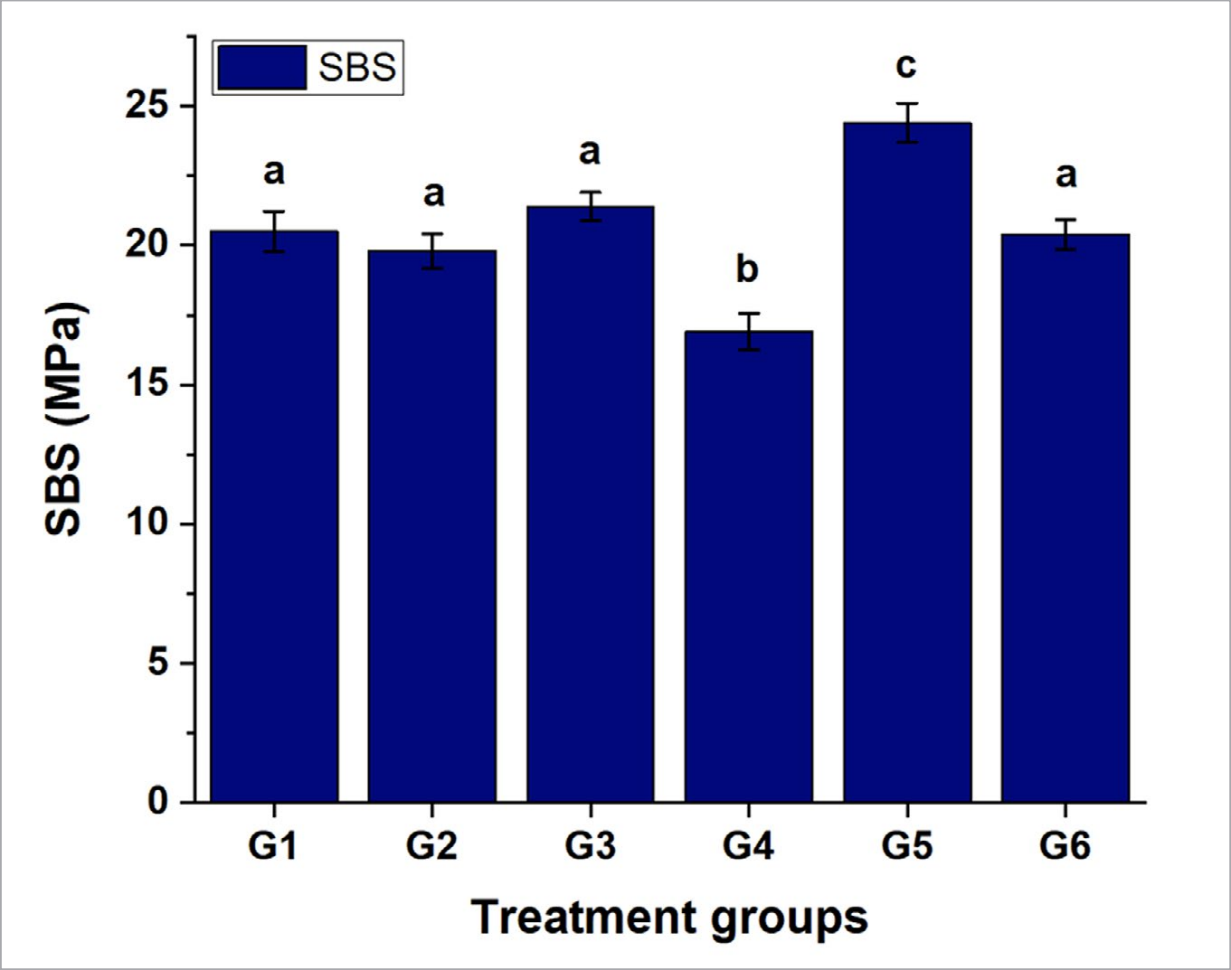
Shear bond strength (SBS, MPa) results across treatment groups (G1–G6).

Figure 2 shows the shear bond strengths (SBS) for baseline (G1) and all treated groups (G2–G6). G5 (KI + MA) had statistically significantly higher SBS than did other groups (p ≤ 0.010). This finding indicates that microabrasion after KI application improved the bonding surface and adhesive performance. G4 had the lowest SBS compared to the other groups (p ≤ 0.018), suggesting that bleaching after KI treatment may hinder resin infiltration or compromise the enamel surface. Groups G1 (baseline control), G2, G3, and G6 did not differ statistically significantly (p ≥ 0.459), indicating comparable bonding performance within this subset. Overall, the SBS values were in descending order: G5 > G3 > G1 > G6 > G2 > G4, as summarized in Table 3, confirming that KI combined with microabrasion produces the best bonding conditions, while bleaching after KI treatment has the most negative impact on bond strength.

**Table 3:** Shear bonding strength (MPa) means (standard deviation) after the designated treatments

Groups	SBS (MPa)
G1	20.5 (3.00)^a^
G2	19.8 (2.57)^a^
G3	21.4 (2.06)^a^
G4	16.9 (2.69)^b^
G5	24.4 (2.87)^c^
G6	20.4 (2.18)^a^
Lowercase letters indicate statistically significant difference between treatments (p < 0.05).	

## DISCUSSION

The application of conservative approaches, including KI, bleaching, microabrasion, and their combinations, statistically significantly affected the average surface roughness and SBS of s-RCLs treated with SDF. Consequently, the null hypothesis was rejected. This study simulated a clinical scenario involving patients treated with SDF pursuing minimally invasive interventions (e.g., bleaching, microabrasion) to improve the appearance of SDF-induced black stains. We aimed to address two primary concerns: first, the potential impact of successful esthetic improvement on the surface roughness of treated enamel, and second, the implications of subsequent restorative procedures, should residual staining necessitate masking, particularly concerning the potential effects on bonding efficacy to the modified tooth substrate. Therefore, an in-vitro model of remineralized caries-like lesions (s-RCLs) was utilized from a previously established model, with standardized steps for demineralization, staining, and remineralization.^7,8^ Furthermore, this study focused on the inclusion of a saturated KI solution in all treatment groups (excluding controls). The role of KI is to chemically neutralize and minimize the formation of silver phosphate, thereby reducing dark stains in s-RCLs to a lighter, more easily targeted precipitate by other treatments. This process is a crucial first step that cannot be achieved through microabrasion or bleaching alone.^21,22^

The non-statistically-significant changes in baseline Ra values among groups confirmed consistent and effective preconditioning of sound specimens, enabling subsequent analyses. The baseline surfaces were the smoothest, highlighting the importance of preconditioning steps, including grinding, polishing, washing, and storage. Artificial caries-like lesions were induced using an acidic medium to mimic the bacterial environment which causes the dissolution of enamel minerals. The demineralization process was regulated by incorporating Ca_2_^+^ and PO_4_^3-^ ions, and carboxymethylcellulose to promote uniformity of ions and adhesion on the lesion surface, thus better simulating oral conditions. Likewise, all groups exhibited a comparable Ra increase after demineralization, indicating uniform mineral loss and similar lesion-like surfaces. Within each group, the demineralized surface became statistically significantly rougher than the baseline, reflecting the development of porosity due to mineral loss and surface erosion.

The increase in Ra in G2 after SDF treatment is attributed to the accumulation of silver-based precipitates (e.g., metallic silver, silver oxide, or silver phosphate), which form an insoluble layer that occludes the enamel pores while the excess increases the surface roughness. However, the change in Ra was not statistically significant, suggesting that only a limited amount of silver-based solids accumulated on the surface. This case affirms sufficiently balanced silver content for the remineralizing process. The immediate addition of KI to 38% SDF-treated enamel induces a reaction with the excess silver-free ions to form a creamy white precipitate of silver iodide.^17,23,25^ In addition, G3 exhibited a slightly smoother surface than G2, likely due to KI’s hygroscopic nature, which reduces the surface tension of SDF, enhances solubility, and improves its penetration into the tooth structure. These actions alter the rate of silver ion formation, potentially leading to fewer particles on the surface and, thus, less roughness.^17,26^ Within the treatment groups, no statistically significant differences were observed among G1, G2, and G3, consistent with previously reported findings.^22^ However, the benefit of KI may diminish over time due to the instability of AgI under light and temperature, leading to the formation of silver and iodine, which subsequently causes darkening of the treated area.^10^ To address this limitation, the SDF-KI-treated groups underwent further conservative treatments, including at-home bleaching, microabrasion, and their combination.

To achieve the desired effect, dental bleaching was performed using 15% CP (4.5% hydrogen peroxide) for 4 h daily over seven days. This concentration is associated with reduced cytotoxicity to human pulp as well as minimal discomfort, making it suitable for at-home application^10,11^ G4 exhibited statistically significantly higher roughness than G3, likely due to the bleaching agent’s acidity and its low molecular weight, which facilitated enamel penetration and enhanced the release of reactive oxygen and free radicals that effectively bleached the stained areas. This consequently increases surface porosities, depression, and irregularities.^7,31^ The highest statistically significant Ra value was observed for microabrasion treatment in G5, and is attributed to the two key components in microabrasion. The abrasive chemical action of 6.6% HCl and the mechanical motion of the rubber cup across the surface simultaneously eroded and abraded the enamel surface.^28,30^ However, this increase was effectively reduced with subsequent bleaching treatment, as observed in group G6. Microabrasion may introduce superficial irregularities, such as microporosities, craters, and disrupted enamel prisms. These irregularities appear susceptible to peroxide oxidation, which may facilitate surface leveling through oxidative breakdown, leading to a measurable change in morphology toward a smoother enamel surface.^13,19^

The SBS was evaluated using a nanohybrid resin-based composite (Filtek Z350, 3M ESPE), selected for its widespread clinical use.^9^ Among all groups, microabrasion (G5) was statistically significantly the most effective surface treatment for improving the bond strength to composite resin (24.4 MPa). This enhancement is attributed to the dual-action chemo-mechanical mechanism, increasing the surface area and creating a rough, microporous surface that enhances micromechanical interlocking via resin tags, consistent with the Ra findings.^15,24^ Furthermore, previous studies analyzing surface morphology support our findings, suggesting that the microabrasion technique sufficiently removes the superficially weakened enamel substrate and silver residue to expose intact, sound enamel, which is suitable for optimal adhesive bonding.^18,30^ Despite a two-week post-bleaching delay intended to dissipate residual bleaching agents and facilitate enamel rehydration, G4, which underwent at-home bleaching and presented with elevated surface roughness, demonstrated the lowest SBS, a statistically significant bond strength reduction among the test groups (16.9 MPa). The decrease in adhesion can be attributed to the enamel’s poor prism-etching pattern, altered mineral content, or residual oxygen radicals, which may interfere with the diffusion of the bonding agent, affect the polymerization, and hinder the formation of resin tags crucial for micromechanical retention.^3^ The intermediate SBS in G6 (20.4 MPa) reflects a balanced effect of the two treatments; this superior SBS in G6 compared to G4 is a result of effective enamel resurfacing. In G4, bleaching created a structurally weakened substrate manifested in a collapse and loss of prismatic enamel, which is not easily penetrated by the resin. Additionally, silver particles on the surface are a physical barrier, which collectively compromises resin infiltration and bonding. However, the chemical/mechanical action of microabrasion in G6 removed the compromised superficial enamel layer (25–200 μm) and silver particles. This preparation exposed an underlying uniform enamel structure that can be easily penetrated by the bleaching agent, resulting in a highly rough surface that maximizes micromechanical retention and resin infiltration depth, thereby significantly increasing its SBS. Furthermore, G1, G2, G3, and G6 (19.8-21.4 MPa) demonstrated minute variations in SBS, with no statistically significant differences among them, in agreement with a previous study.^4^ Notably, G3 was slightly superior, possibly due to KI enhancing SDF penetration and reducing pore occlusion, thereby improving bonding.

There are a few limitations inherent in the current study. For instance, its in-vitro nature may not fully reflect the complexities of the oral environment and clinical conditions. Microabrasion using 6.6% HCl and at-home bleaching with 15% CP offer esthetic benefits and are widely considered safe under professional supervision.^14,30^ However, unsupervised or prolonged application, particularly in young or elderly patients, may lead to postoperative sensitivity and alter the enamel structure (e.g., reduce its microhardness). Consequently, a postoperative remineralization strategy, such as topical fluoride applications, should be implemented.^14^ Additionally, the use of specific treatment concentrations and application times in each treatment, as well as sample variability, may limit the generalizability of the results to other clinical protocols. Therefore, clinical extrapolation of the findings should be approached with caution.

Nevertheless, this study demonstrated that a minimally invasive approach may offer a promising strategy for treating s-RCLs-KI, particularly when yielding low surface roughness and enhanced SBS. Stand-alone at-home bleaching increased surface roughness and diminished SBS, whereas microabrasion alone enhanced the SBS; however, the statistically significant elevation in Ra raises concerns regarding its susceptibility to plaque accumulation, facilitating recurrent caries, and potential staining. Notably, the combined application of KI, bleaching, and microabrasion on SDF-treated lesions exhibited synergistic benefits, as it had the least statistically significant increase in roughness and maintained an acceptable average SBS, suggesting a superior functional outcome compared to microabrasion or bleaching as individual therapies.

## CONCLUSIONS

The synergistic combination of potassium iodide (KI), microabrasion (6.6% HCl), and at-home bleaching (15% CP for one week) offered superior conservative treatment for SDF-treated s-RCL. Importantly, the combination treatment outperformed microabrasion or bleaching alone, which were associated with excessive surface roughening or poor bonding. The findings show that KI neutralizes discoloration without affecting bonding, microabrasion smooths and renews the enamel surface, and subsequent bleaching improves esthetics while maintaining functional bonding capacity. Clinically, this multimodal approach strikes a balance between esthetic improvement and adhesive performance, making it a promising protocol for the restorative management of SDF-treated lesions. Future in-vivo studies and long-term follow-ups are needed to confirm the durability, color stability, and patient-reported outcomes of this combined treatment in routine dental care.

## ACKNOWLEDGMENT

The authors extend their appreciation to the Ongoing Research Funding program – Research Chairs (ORF-RC-2025-5100).
